# A Novel Approach Based on Real-Time PCR with High-Resolution Melting Analysis for the Simultaneous Identification of *Staphylococcus aureus* and *Staphylococcus argenteus*

**DOI:** 10.3390/foods13183004

**Published:** 2024-09-22

**Authors:** Daniele Chieffi, Dafne Bongiorno, Anna Licitra, Floriana Campanile, Vincenzina Fusco

**Affiliations:** 1 Institute of Sciences of Food Production, National Research Council of Italy (CNR-ISPA), 70126 Bari, Italy; daniele.chieffi@ispa.cnr.it (D.C.); anna.licitra@ispa.cnr.it (A.L.); 2 Medical Molecular Microbiology and Antibiotic Resistance Laboratory (MMARLab), Department of Biomedical and Biotechnological Sciences (BIOMETEC), University of Catania, 95123 Catania, Italy; d.bongiorno@unict.it (D.B.); f.campanile@unict.it (F.C.)

**Keywords:** *Staphylococcus aureus*, *Staphylococcus argenteus*, *Staphylococcus aureus* complex, real-time PCR, high-resolution melting, HRM, superoxide dismutase, *sodA* gene, species-specific PCR

## Abstract

*Staphylococcus* (*S*.) *aureus* is a pathogenic bacterium able to cause several diseases in humans and animals as well as foodborne intoxications. *S. argenteus*, being phenotypically and genotypically related to *S. aureus*, is part of the so-called *S. aureus* complex and recently recognized as an emerging pathogen able to cause, like *S. aureus*, several diseases both in humans and animals, and foodborne poisoning outbreaks. However, it has been reported that the widely used conventional PCR of Brakstad et al. [*Journal of Clinical Microbiology*, *30*(7), 1654–1660, (1992)] targeting the thermostable nuclease gene may provide false-positive *S. aureus*, as it is able to amplify also *S. argenteus*. Here, we developed a novel two-step approach that, following the PCR of Brakstad et al. (1992), discriminates *S. aureus* from *S. argenteus* by a real-time PCR with high-resolution melting analysis (rt-PCR-HRM). In particular, targeting a polymorphic 137 bp region of the *sodA* gene, our developed rt-PCR-HRM method clearly discriminated *S. aureus* from *S. argenteus*, showing a remarkable difference in their amplification product melting temperatures (approximately 1.3 °C) as well as distinct melting curve shapes. The good sensitivity, reproducibility, user friendliness, and cost effectiveness of the developed method are advantageous attributes that will allow not only its easy employment to correctly identify misidentified isolates present in various collections of *S. aureus,* but also expand the still lacking knowledge on the prevalence and distribution of *S. argenteus*.

## 1. Introduction

*Staphylococcus* (*S*.) *aureus* (phylum *Bacillota*; class *Bacilli*; order *Bacillales*; family *Staphylococcaceae*; genus *Staphylococcus*) (https://www.ncbi.nlm.nih.gov/Taxonomy/Browser/wwwtax.cgi?id=1280, accessed on 1 March 2024) is a Gram-stain-positive, facultatively anaerobic, coagulase- and catalase-positive coccoid bacterium discovered in the late 1800s thanks to the Scottish surgeon Sir Alexander Ogston, who observed masses that “looked like bunches of grapes” in pus from a surgical abscess (1880), and the German physician Friedrich Julius Rosenbach, who named *S. aureus* for its characteristic yellow colonies (from the Latin aurum, gold) (1884) [1].

*S. aureus* is a pathogenic microorganism able to cause a wide variety of systemic and localized diseases, including bacteremia, toxic shock syndrome, endocarditis, epidural abscess, meningitis, septic thrombophlebitis, as well as osteoarticular, pleuropulmonary, soft tissue, skin, urinary tract, and prosthetic device-related infections [2,3]. It also poses a threat to food safety, causing staphylococcal food poisoning (SFP) [4] due to its ability to produce 33 known staphylococcal enterotoxins (SEs) and enterotoxin like-toxins (SEls) [5,6]. This foodborne disease is characterized by severe nausea and projectile vomiting, while other symptoms comprise retching, diarrhea, abdominal cramps, headache, muscle ache, dizziness, shivering, and general weakness sometimes associated with a moderate fever [7,8]. Although it is usually a self-limiting illness lasting from several hours to 48 h, it has often an abrupt and severe onset after a short incubation period (30 min to 8 h following the ingestion of the contaminated food), and in severe cases, dehydration, prostration, and shock can occur, requiring hospitalization [7,8]. In particular, deaths can range from 0.03% in healthy people to 4.4% in infants, elderly, or patients with underlying chronical illness [8].

In 2015, two novel species, i.e., *S. argenteus* and *S. schweitzeri*, which are phenotypically and genotypically related to *S. aureus*, were identified, and the *S. aureus*-related complex was described [9] and then also enlarged to include two other species (i.e., *S. singaporensis* and *S. roterodami*), although, due to phylogenetic analyses, their identification as separate species remains questionable [10,11,12].

While *S. schweitzeri* has not been associated with pathological conditions and was essentially reported in few animal species on the African continent [13], *S. argenteus* is increasingly recognized as an emerging pathogen [14] able to cause different pathological conditions in humans (e.g., lymphadenitis, endocarditis, joint infection, and sepsis) and animals (e.g., wound infection, abscess, and mastitis in cattle) [15,16,17,18,19,20,21]. In addition, due to its SE-producing ability [22], *S. argenteus* has recently been reported as the causative agent of several foodborne poisoning outbreaks [22,23,24].

It should be emphasized that Brakstad et al. [25] developed a conventional polymerase chain reaction (PCR) assay for *S. aureus* identification by targeting a region of the *S. aureus* thermostable nuclease encoding gene (*nucA*). Currently, this assay is widely used as a standard confirmatory marker for *S. aureus*, although it may also yield positive results for *S. argenteus* (while negative results are obtained for *S. schweitzeri*) [9], actually impairing the species specificity of such method.

As also highlighted by other authors [26,27], the development of novel PCR approaches to correctly identify *S. aureus* and *S. argenteus* may therefore represent a great asset, in particular to assess the actual prevalence and spreading of *S. argenteus* [26] and overcome the limitations of the conventional *nucA* PCR of Brakstad et al. [25].

Among the PCR techniques, real-time PCR with high-resolution melting analysis (rt-PCR-HRM) has the potential to discriminate different bacterial species based on the nucleotide differences in their DNA sequences. In essence, rt-PCR-HRM consists of the melt curve analysis of the DNA fragments obtained through a PCR amplification, typically performed with a HRM-compatible real-time PCR instrument. In particular, it employs a double-stranded nucleic acid-specific fluorescent dye (e.g., LCGreen, EVAGreen, or SYTO9) [28] able to saturate all DNA binding sites during the DNA amplification, with the application of narrow temperature increments during the subsequent melt curve stage. Thereafter, the analysis of the resulting melting data allows the differentiation of the obtained PCR amplicons based on their nucleotide sequence, length, and GC content. Overall, the affordability of reagents, the utilization of instrumentation now found in many laboratories, and the simplicity of the approach constitute the main advantages of the HRM technology [28]. Considering the described scenario, in this study, a novel rt-PCR-HRM approach was developed to simultaneously identify and discriminate *S. aureus* and *S. argenteus*, overcoming the limitations of the conventional *nucA* PCR of Brakstad et al. [25]. To test the validity of the protocol, we carried out the study in two different research facilities (CNR-ISPA, Bari, Italy; and MMARLab, University of Catania, Catania, Italy) with different isolates, DNA extraction methods, and real-time PCR instruments.

## 2. Materials and Methods

### 2.1. Reference Strains and Bacterial Isolates

DNA aliquots from 87 foodborne *S. aureus*, isolated and characterized in previous studies conducted by our group [2,4,29,30], were used at CNR-ISPA (Bari, Italy) to develop the real-time PCR HRM assay (Table 1). Such *S. aureus* isolates were formerly identified by conventional species-specific PCR utilizing the primer set targeting the 270 bp region of the *nucA* gene of *S. aureus* [25]. DNA aliquots of *S. aureus* (DSM 20231^T^) and *S. argenteus* (DSM 28299^T^) type strains were included in each PCR run to serve as positive controls as well as reference strains for species assignment during the analysis. Moreover, DNA aliquots of a *S. schweitzeri*-type strain (DSM 28300^T^) were used during the preliminary stages of the assay development to test the ability of the method to also discriminate *S. schweitzeri*. DNA aliquots of *Listeria* (*L.*) *monocytogenes*-type strain (DSM 20600^T^), *L. monocytogenes* LMG 23191, and *Campylobacter curvus* LMG 11310 were included as negative controls to assess the absence of any nonspecific amplification.

At the MMARLab (University of Catania, Catania, Italy), 22 clinical *S. aureus* isolates previously characterized by conventional species-specific molecular methods [31] were further tested (Table 2) to verify the applicability of the developed method. DNA aliquots of *S. aureus* (ATCC 29213), *S. argenteus* (DSM 28299^T^), and *S. schweitzeri* (DSM 28300^T^) were included in PCR runs to serve as reference strains. Additionally, one *S. argenteus* strain (*S. argenteus* S1 2021, belonging to the MMARLab collection) and seven ATCC coagulase-negative staphylococci (CoNS) (ATCC Microbe products—https://www.atcc.org/) were used as further controls (Table 2).

### 2.2. Bacterial Growth and DNA Extraction

The bacterial isolates were grown in brain hearth infusion (BHI) broth (Oxoid, Basingstoke, UK) with the addition of 0.6% yeast extract (Biolife Italiana, Milano, Italy), incubated at 37 °C for 24 h. Then, aliquots of broth cultures were used for the DNA extraction.

At ISPA-CNR, the DNA of *S. aureus* DSM 20231^T^, *S. argenteus* DSM 28299^T^, *S. schweitzeri* DSM 28300^T^, *L. monocytogenes* DSM 20600^T^, *L. monocytogenes* LMG 23191, and *C. curvus* LMG 11310 as well as of 60 *S. aureus* isolates (Table 1) was extracted using an alcohol-based DNA extraction method referred to as the “classical method”. Briefly, 1 mL broth culture aliquots were centrifuged at 8000 rpm for 10 min, and the resulting bacterial pellets were washed using 1 mL of sterile SSC buffer (0.15 M NaCl and 15 mM sodium citrate, pH 7.00) and 1 mL of TE buffer (10 mM Tris-HCl and 1 mM EDTA, pH 8.00), respectively, and then resuspended with 500 µL of TE buffer. The cell lysis was performed adding lysostaphin (2 mg/mL solution) (Sigma-Aldrich, St. Louis, MO, USA) for *Staphylococcus* spp. or lysozyme (50 mg/mL solution) (Sigma-Aldrich, St. Louis, MO, USA) for the other species, incubated at 37 °C for 1 h. RNA was degraded by the addition of RNaseA (100 mg/mL solution) (Qiagen, Milan, Italy), followed by incubation at 25 °C for 1h. Then, sodium dodecyl sulfate (25% solution) (Sigma-Aldrich, St. Louis, MO, USA) and proteinase-K (≥800 units/mL solution) (Sigma-Aldrich, St. Louis, MO, USA) were added and incubated at 37 °C for 1.5 h. Proteins were precipitated by addition of Protein Precipitation Solution (Promega Italia, Milan, Italy) followed by centrifugation at 16,000× *g* for 5 min at 4 °C. After the supernatant recovery, the DNA was precipitated and then washed by two centrifugations at 16,000× *g* for 10 min at 4 °C, first adding isopropyl alcohol to the supernatant and then adding 70% ethanol solution to the obtained DNA pellet. Finally, the DNA was resuspended in sterilized ultrapure water. The quantity and purity of the extracted DNA was assessed by spectrophotometric method using NanoDrop ND-1000 (Thermo Fisher Scientific, Waltham, MA, USA).

The DNA of another 27 *S. aureus* isolates (Table 1) was also extracted by a simple boiling method, henceforth referred to as the “rapid method”, using a Chelex-based reagent (InstaGene Matrix, Bio-Rad, Hercules, CA, USA) as reported by Mekhloufi et al. [30]. Briefly, 500 µL broth culture aliquots were centrifuged at 12,000 rpm for 90 s, and the resulting pellet was washed using 1 mL of sterile distilled water. DNA was extracted adding 200 µL of InstaGene Matrix to the bacterial cell pellet following the manufacturer’s instruction.

At the MMARLab, genomic DNA for real-time PCR amplification (Table 2) was extracted using the QIAamp^®^ DNA Mini Kit (cat. No. 51306, Qiagen, Milan, Italy), with some modifications to the manufacturer’s protocol. In brief, bacterial suspensions were centrifuged and resuspended in 200 μL of 0.9% physiological saline solution, and then, they underwent two cycles of freezing and thawing. Following centrifugation, the bacterial pellet was resuspended in 180 µL of an enzyme solution containing 20 mg/mL lysozyme (cat. No. 10837059001, Sigma-Aldrich, Merck KGaA, Darmstadt, Germany) and 100 μg/mL lysostaphin (cat. No. L7386-15MG, Sigma-Aldrich, Merck KGaA, Darmstadt, Germany) in Tris-EDTA (TE) buffer, pH 8.0 (cat. No. AM9849, Ambion, Invitrogen, Waltham, MA, USA). Aside from these adjustments, the manufacturer’s instructions were followed [32]. For ATCC strains, DNA extraction was performed using the Pitcher protocol [33]. DNA quantification was carried out using Quanti-it Assays via fluorimetric analysis on a Qubit 2.0 (Invitrogen—Life Technologies, Waltham, MA, USA), following the manufacturer’s guidelines.

### 2.3. Primers and Real-Time PCR-HRM Conditions

A pair of primers was designed to amplify a region of the *sodA* gene encoding the superoxide dismutase (manganese-dependent metalloenzyme) of *S. aureus* and *S. argenteus*.

**Figure 1:**
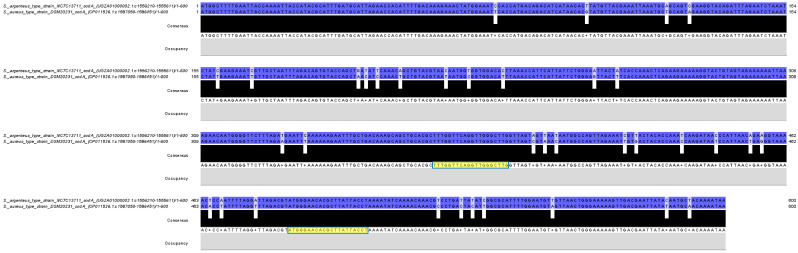
Alignment of the *sodA* genes of *S. aureus* DSM 20231^T^ and *S. argenteus* NCTC 13711^T^, obtained by Clustal Omega [34] and visualized in Jalview [35]. Highlighted in yellow are the annealing sites of the forward primer sodAaur-argF (5′-TTTGGTTCAGGTTGGGCTTG-3′) and the reverse primer sodAaur-argR (5′-AGGTAATAAGCGTGTTCCCAT-3′).

For this purpose, the *sodA* gene sequences of *S. aureus* DSM 20231^T^ (accession number: CP011526.1) and *S. argenteus* NCTC13711^T^ (accession number: UGZA01000002.1), available in GenBank (https://www.ncbi.nlm.nih.gov/genbank/) were aligned using the Clustal Omega program [34] and visualized using Jalview software (v. 2.11.3.2) [35]. The aligned sequences were analyzed to identify a polymorphic region useful for primer design, using the Primer3 software (v. 4.1.0) [36]. In particular, the forward (sodAaur-argF, 5′-TTTGGTTCAGGTTGGGCTTG-3′) and the reverse (sodAaur-argR, 5′-AGGTAATAAGCGTGTTCCCAT-3′) primers were designed to target a 137 bp region of the *sodA* gene comprising 12 mismatches between the reference sequences, as shown in Figure 1.

At CNR-ISPA, the DNA amplification was performed in the 7500 Fast Real-Time PCR System (Applied Biosystems, Waltham, MA, USA). Amplification data were analyzed using the SDS software (v. 1.4) (Applied Biosystems, Waltham, MA, USA) and HRM analysis of the melting curves was performed using the HRM software (v. 2.0) (Applied Biosystems, Waltham, MA, USA). The PCR reaction mixture was prepared as follows: 10 µL of MeltDoctor™ HRM Master Mix (Applied Biosystems Waltham, MA, USA), 0.2 µM of each primer, 10 ng of DNA extracted using the “classical method”, or 1 µL of DNA extracted using the “rapid method”. Finally, sterilized ultrapure water was added to reach the total volume of 20 µL. No template controls (NTCs) were included in each PCR run, with sterilized ultrapure water used instead of DNA in the reaction mixture, to verify the absence of any nonspecific signals. The DNA was amplified by applying the following thermocycling conditions: initial denaturation at 95 °C for 10 min, 40 cycles of denaturation at 95 °C for 15 s, and annealing/extension at 60 °C for 1 min. In order to obtain the melting curves of the amplification products, a dissociation step was performed by applying the following conditions: denaturation at 95 °C for 15 s, annealing at 60 °C for 1 min, high resolution melting at 95 °C for 15 s, and annealing at 60 °C for 15 s.

The obtained melting curves were analyzed, and the normalized melt curves plot and the difference plot were generated. In particular, both plots were obtained after the normalization of the fluorescence levels by setting the pre-melt and post-melt values on the derivative melt curves in order to scale the signal in the range from 100 (pre-melt) to 0% (post-melt).

To assess the repeatability of the developed rt-PCR-HRM assay, the intra- and inter-assay coefficient of variation (CV%) of the melting temperatures (Tm) of a subset of 19 isolates was analyzed. For this purpose, the DNA of these isolates was tested in triplicate in two independent runs, performed on two different days.

At the MMARLab, further real-time PCR-HRM assays were performed on a Rotor-Gene Q 5plex platform (Qiagen, Milan, Italy) in 10 μL reaction mix including the following: 1X Type-it HRM PCR Kit master mix (Qiagen, Milan, Italy), 0.2 μM of each primer, and 1 μL of template DNA (10 pg/μL). The cycling conditions included a pre-denaturation step of 10 min at 95 °C, 40 annealing cycles for 1 min at 60 °C, and denaturation for 15 s at 95 °C, with the acquisition of fluorescence data on the green channel. The amplimers were subjected to progressive thermal denaturation from 60 °C for 1 min and at 95 °C for 15 s, with a temperature increase of 1.6 °C/s.

### 2.4. Limit of Detection and Efficiency of the Assay

In order to evaluate the sensitivity of the developed rt-PCR-HRM assay, the limit of detection (LOD) was assessed by testing in triplicate, in a range between 10^7^ and 10^0^ genome equivalents (GE), decimal serial dilutions of the DNA of each reference strain (*S. aureus* DSM 20231^T^ and *S. argenteus* DSM 28299^T^) using the 7500 Fast Real-Time PCR System (Applied Biosystems, Waltham, MA, USA). Specifically, with an estimated genome size of 2.8 Mb [37], the amount of 2.86 fg of DNA was calculated as corresponding to 1 GE for *S. aureus* and *S. argenteus* [38]. The LOD was determined as the lowest DNA dilution in which triplicate amplification and concomitant correct species identification occurred.

In order to calculate the efficiency of the developed assay, standard curves were constructed, and slope and R^2^ values were determined. The assay efficiency (E%) was calculated according to the following formula: [10^(−1/slope)^ − 1] × 100.

## 3. Results

### 3.1. HRM Analysis of the Tested Isolates

**Figure 2:**
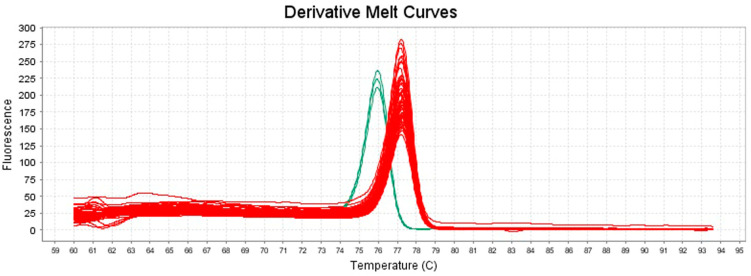
Derivative melt curves generated by HRM analysis of the amplification products of 19 representative isolates tested in triplicate within one intra-assay reproducibility test. In red are shown the derivative melt curves of strains identified as *S. aureus* and the reference strain *S. aureus* DSM 20231^T^; in green are shown the derivative melt curves of the reference strain *S. argenteus* DSM 28299^T^ (ISPA-CNR).

At ISPA-CNR, for all the *S. aureus* isolates tested in the rt-PCR-HRM assay (*n* = 87), the amplification products and the corresponding melting curves were obtained, while no amplifications occurred for the negative controls. Specifically, the mean melting temperatures (Tm) and the corresponding melting curves (derivative melt curves) of the representative subset isolates (n = 19) and reference strains (*S. aureus* DSM 20231^T^ and *S. argenteus* DSM 28299^T^), tested in triplicate and used for the intra- and inter-assay reproducibility, are shown in Table 3 and Figure 2.

The reference strain *S. argenteus* DSM 28299^T^ exhibited a mean Tm of 75.93 ± 0.03 °C (Table 3), and the reference strain *S. aureus* DSM 20231^T^ exhibited a mean Tm of 77.22 ± 0.04 (Table 3). The isolates identified as *S. aureus* showed a mean Tm of 77.28 ± 0.13 °C (Table 3) similar to the reference strain *S. aureus* DSM 20231^T^. Thus, the HRM assay displayed a remarkable difference in mean Tm between *S. aureus* and *S. argenteus* that was approximately of 1.3 °C.

**Figure 3:**
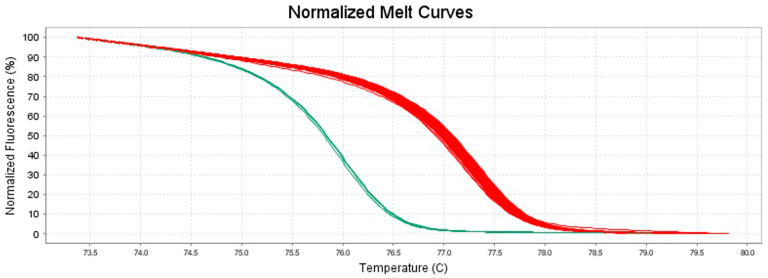
Normalized melt curves generated by HRM analysis of the amplification products of 19 representative isolates tested in triplicate within one intra-assay reproducibility test. In red are shown the normalized melt curves of strains identified as *S. aureus* and the reference strain *S. aureus* DSM 20231^T^; in green are shown the normalized melt curves of the reference strain *S. argenteus* DSM 28299^T^ (ISPA-CNR).

**Figure 4:**
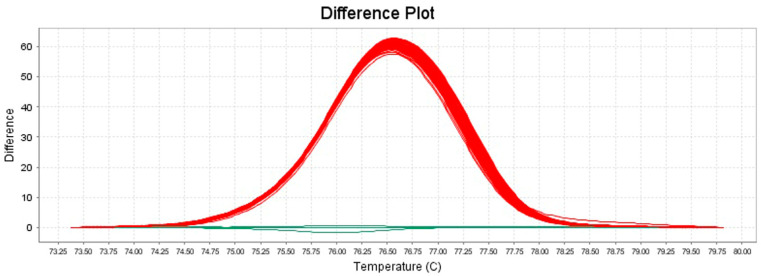
Difference plot generated by HRM analysis of the amplification products of 19 representative isolates tested in triplicate within one intra-assay reproducibility test. In red are shown the melt curves of strains identified as *S. aureus* and the reference strain *S. aureus* DSM 20231^T^; in green are shown the melt curves of the reference strain *S. argenteus* DSM 28299^T^ (ISPA-CNR).

Furthermore, the behavior of the melting curves observed in the normalized plot (Figure 3) and difference plot (Figure 4) also allowed a clear differentiation of species between *S. aureus* and *S. argenteus*. Otherwise, no such clear differentiation was observed for *S. schweitzeri*, whose mean Tm value and melt curves were very similar to those of *S. aureus* (Figure S1 and Table S1).

The *S. aureus* strains whose DNA was extracted by the rapid boiling method and the *S. aureus* strains whose DNA was extracted by the classical method showed similar mean Tm values of 77.16 ± 0.05 and 77.34 ± 0.11 °C, respectively (Table 3), as well as overlapping melting curve trends in all plots considered (Figure 2, Figure 3 and Figure 4). Thus, the developed method allowed the identification of the isolates independently of the DNA extraction method used.

**Figure 5:**
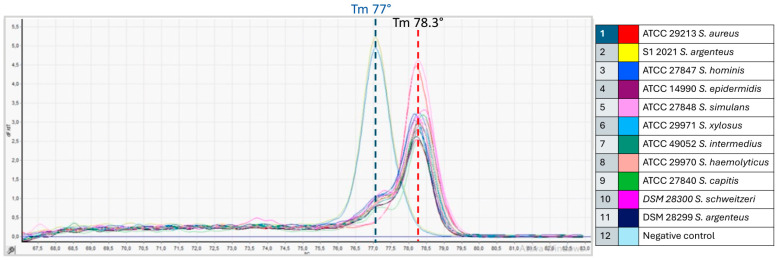
Derivative melt curves generated by HRM analysis of the amplification products of 11 representative *Staphylococcus* spp. isolates tested in triplicate within one intra-assay reproducibility test. The red line shows the average melting temperature (Tm) of strains identified as *S. aureus* and CoNSA; in blue are the average melting temperature (Tm) values of the reference strain *S. argenteus* DSM 28299^T^ and internal control *S. argenteus* S1 2021 (MMARLab).

At the MMARLab, the reference strains of *S. argenteus* DSM28299^T^, *S. aureus* ATCC 29213, and *S. schweitzeri* DSM20300^T^ were first analyzed by comparing them with the internal control *S. argenteus* S1 2021 and seven ATCC coagulase-negative staphylococci. It emerged that the melting temperature of *S. argenteus* DSM28299^T^ and S1 2021 was 77 °C for both strains (Figure 5), while that of *S. aureus, S. schweitzeri,* and the seven coagulase-negative staphylococci varied between 78.3 °C and 78.5 °C (Figure 5). It allowed rapid identification of *S. argenteus* from the other tested staphylococcal species, but on the other hand, the HRM analysis, showing peaks at very similar melting temperatures, could not be used to discriminate *S. aureus* from *S. schweitzeri* and the other coagulase-negative staphylococcal species.

**Figure 6:**
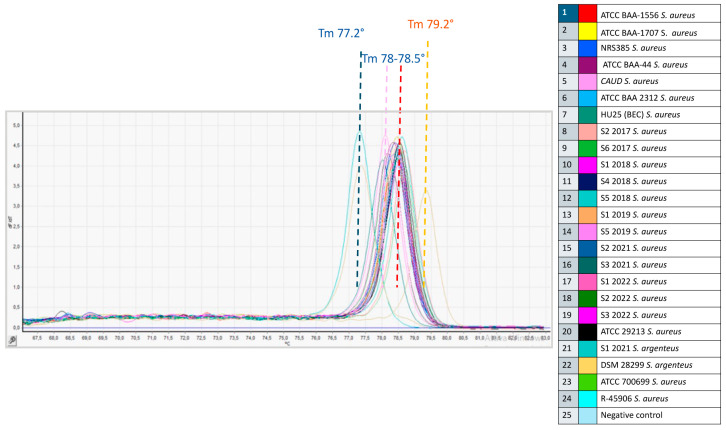
Derivative melt curves generated by HRM analysis of the amplification products of 22 *S. aureus* and 2 *S. argenteus* isolates tested in triplicate within one intra-assay reproducibility test. The red/pink lines show the average melting temperatures (Tm) of strains identified as *S. aureus*; the blue line shows the average melting temperature (Tm) of the reference strain *S. argenteus* DSM 28299^T^ and internal control *S. argenteus* S1 2021; the yellow line shows the Tm of the S1 2019 strain previously misidentified as *S. aureus* (MMARLab).

After acquiring the melting temperatures (Tm) of *S. argenteus* compared to the other *Staphylococcus* spp., we proceeded with the analysis of all the *S. aureus* clinical isolates (including MRSA and MSSA) belonging to several international clones, as shown by their respective STs (Table 2). For this purpose, their respective Tm values were compared with those of *S. aureus* ATCC 29213 as well as *S. argenteus* DSM 28299^T^ and S1 2021, which were used as reference control strains. Also, in this case, it was possible to confirm an almost identical Tm of 77.2 °C for both isolates of *S. argenteus*, whose peaks overlapped and were remarkably distant from those of the entire *S. aureus* group, with Tm values between 78 and 78.5 °C. Also, the DNA of the ATCC strains extracted with two different methods confirmed the same melting curves (Figure 6).

An unexpected result emerged from the S1 2019 strain, which showed a Tm of 79.2 °C, highlighted by the peak spaced from all the other isolates (Figure 6). This result, which deserves further investigation such as gene sequencing, could represent a variant of the nucleotide sequence of the *sodA* gene and suggests that it is a different species than *S. aureus*, which was previously attributed to this isolate. This result also highlights how HRM analysis is significantly more effective in identifying species than other methods, especially phenotypic ones.

### 3.2. Limit of Detection

The lowest amount of DNA at which the amplification of triplicates occurred with concomitant correct species identification was 10 genome equivalents (GE) for *S. aureus* (Table 4, Figure S2a) as well as for *S. argenteus* (Table 4, Figure S3a).

### 3.3. Standard Curves and Efficiency

The standard curves generated with serially diluted DNA for *S. aureus* DSM 20231^T^ and *S. argenteus* DSM 28299^T^ showed an R^2^ of 0.997 and 0.995 (Figures S2b and S3b), with an amplification efficiency of 103% and 105%, respectively.

## 4. Discussion

Considering the implications for human and animal health, it is of crucial importance to discriminate and identify *S. aureus* and *S. argenteus* in order to (i) know the etiological agent in case of disease; (ii) assess the prevalence of these two microorganisms in humans, animals, as well as in food; and (iii) expand the still-lacking knowledge on the presence and distribution of *S. argenteus*.

In 2015, a first approach for the identification of *S. aureus* and *S. argenteus* was developed by the utilization of the matrix-assisted laser desorption ionization–time of flight mass spectrometry (MALDI-TOF MS) [9], whose discriminatory ability was based on the different cellular peptide/protein profiles exhibited by the two species. Although specific signals have been identified and utilized in various MALDI-TOF MS protocols [39,40], Becker et al. [41] pointed out that some older MALDI-TOF MS databases do not include reference profiles suitable for the species separation, and moreover, some recent versions of commercially available databases are not properly up-to-date. Taking into account such limitations and considering the costs associated with the application of this method, it is desirable to develop novel identification methods, identifying new species-specific markers allowing the *S. aureus* and *S. argenteus* discrimination.

In this perspective, DNA-based methods with the amplification of target sequences by PCR represent a valuable option, as these techniques are widely used in diagnostic and research laboratories and are relatively economically affordable, robust, and user friendly [42,43,44].

Amplification by PCR and subsequent sequencing of the 16S rRNA gene is a well-established method for the identification of bacterial species, although it is widely recognized that for genetically closely related species, it is not possible to achieve an accurate species identification, in particular when the percentage of similarity of the 16S rRNA gene nucleotide sequence is higher than 97%, the value used as a threshold for identification at the species level [45].

Specifically, *S. aureus* and *S. argenteus* have identical nucleotide sequences of the 16S rRNA gene [9]; therefore, the species identification by its sequencing is not applicable, necessitating different species-specific genetic markers.

As mentioned in the introduction, Brakstad and co-workers [25] developed a PCR protocol in 1992 by designing a pair of species-specific primers (primer 1: 5′-GCGATTGATGGTGATACGGTT-3′ and primer 2: 5′-AGCCAAGCCTTGACGAACTAAAGC-3′) targeting a 270 bp region of the *nucA* gene of *S. aureus* [25]. Currently, amplification of this region is widely used as a standard confirmatory marker for the identification of *S. aureus* [9].

Nevertheless, it was reported by Tong and colleagues [9] that *S. argenteus* also gives positive results in the PCR for the *nucA* gene. Specifically, when this assay is performed using aliquots of *S. argenteus* DNA, it is possible to obtain an amplification product of the same size as that obtained for *S. aureus*, despite the presence of one and five mismatches at the annealing sites of the forward and reverse primers on the *S. argenteus nucA* gene, respectively. This impairs the species specificity of the method of Brakstad et al. [25], not allowing, de facto, the accurate identification of these two staphylococcal species.

In this frame, the rt-PCR-HRM herein developed allows to effectively discriminate *S. aureus* from *S. argenteus*. Nevertheless, our method proved unable to differentiate *S. schweitzeri*, and coagulase-negative staphylococci, from *S. aureus*.

For this reason, a two-step approach is proposed to overcome such limitations: First, isolates should be screened by the PCR of Brakstad et al. [25], which gives negative results for *S. schweitzeri* and coagulase-negative staphylococci [9,25]; then, isolates that give positive results upon the PCR of Brakstad et al. [25] could be accurately identified and discriminated as *S. aureus* or *S. argenteus* by the rt-PCR-HRM of the *sodA* gene developed herein.

Importantly, an immediate application of such method, which will benefit the scientific community, is the possibility to screen the various *S. aureus* collections hosted in different research and clinical centers and already identified by the standard PCR of Brakstad et al. [25], allowing the correct identification of *S. aureus* and *S. argenteus*.

Interestingly, rt-PCR-HRM protocols have recently been developed to identify genotypically closely related bacteria. Landolt et al. [46] developed an rt-PCR-HRM assay to identify species within the *Mycobacterium tuberculosis* complex. Specifically, the application of the method, based on the *gyrB* gene polymorphism, allowed the identification of 63 isolates as belonging to *Mycobacterium* (*M*.) *tuberculosis*, *M. microti*, and *M. bovis*/*M. caprae* species. An rt-PCR-HRM assay was also developed for the identification of 11 species within the genus *Staphylococcus* [47], but this method targeted a region of the *16S rRNA* gene which, therefore, cannot be used for the differentiation of *S. aureus* and *S. argenteus*.

Considering the primary objective of this study to clearly discriminate and identify *S. aureus* and *S. argenteus*, the polymorphic sequence of the *sodA* gene (coding for the superoxide dismutase enzyme) that was previously used, through its sequencing, for the identification of species within the *Streptococcus* and *Enterococcus* genera as well as for the identification of coagulase-negative staphylococci [48] was also found to be a useful genetic marker for the differentiation of the two pathogenic species herein considered.

In particular, the alignment of the nucleotide sequences of the *sodA* genes harbored by the type strains of *S. aureus* and *S. argenteus* showed, among the others, 12 closely located mismatches, allowing the design of a primer pair flanking this polymorphic region to obtain an amplification product of 137 bp. It should be emphasized that the amplification product of an rt-PCR-HRM assay should ideally not exceed the size of 150 bp, as longer amplicons could negatively affect the quality of the melting curves and the resolution of the developed method [46].

Owing to the optimal size and the presence of numerous mismatches, the obtained amplicons for *S. aureus* and *S. argenteus* showed highly dissimilar mean Tm values, with a difference of approximately 1.3 °C (as shown in the intra- and inter-assay reproducibility test), as well as clearly distinguishable shapes of their respective melting curves, as observed especially in the normalized and difference plots, confirming the ability of our assay to clearly discriminate between the two considered bacterial species.

The limit of detection of 10 GE for both *S. aureus* and *S. argenteus* indicates the good analytical sensitivity of the developed method, and in addition, the low coefficient of variation values (CV%), obtained for the intra- and inter-assays demonstrate the good reproducibility of the assay. The latter, observed independently of the method used for DNA extraction of the tested isolates, constitutes an advantageous attribute of robustness and user friendliness of the method, which will be useful for its future applications.

## 5. Conclusions

In this paper, a two-step approach based on the application of the *nucA* PCR of Brakstad et al. [25] and the subsequent utilization of the novel real-time PCR with high-resolution melting analysis targeting a variable region of the *sodA* gene is proposed to allow the simultaneous differentiation and identification of *S. aureus* and *S. argenteus*, overcoming the limitations of the sole *nucA* PCR [25] widely used so far as reference standard for *S. aureus* identification.

The good sensitivity, excellent reproducibility, user friendliness, and relative cost effectiveness of the assay are advantageous attributes that will allow not only its easy employment to correctly identify the isolates that are present in the various collections of *S. aureus* but also confirm the identity of the microorganism in case of disease outbreaks as well as expand, in general, the still-lacking knowledge on the prevalence and distribution of *S. argenteus*, whose increasing relevance as an emerging pathogen is being reported [14].

## Figures and Tables

**Table 1:** Eighty-seven foodborne *S. aureus* isolates used in this study to develop the rt-PCR-HRM assay at CNR-ISPA, Bari, Italy.

Isolate	Origin	Isolate	Origin	Isolate	Origin
178P ^a^	Raw milk	363P ^a^	Raw milk	SA23 ^b^	Minced meat
179P ^a^	Raw milk	372P ^a^	Raw milk	SA33 ^b^	Mashed potatoes
180P ^a^	Raw milk	364P ^a^	Raw milk	SA43 ^b^	Rice
181P ^a^	Raw milk	365P ^a^	Raw milk	SA44 ^b^	Salad
182P ^a^	Raw milk	366P ^a^	Raw milk	SA50 ^b^	Salad
183P ^a^	Raw milk	236P ^a^	Raw milk	SA53 ^b^	Minced meat
184P ^a^	Raw milk	237P ^a^	Raw milk	SA54 ^b^	Beet salad
185P ^a^	Raw milk	238P ^a^	Raw milk	SA58 ^b^	Meat
186P ^a^	Raw milk	239P ^a^	Raw milk	SA73 ^b^	Salad
187P ^a^	Raw milk	240P ^a^	Raw milk	SA78 ^b^	Lentil soup
188P ^a^	Raw milk	241P ^a^	Raw milk	SA83 ^b^	Beans
189P ^a^	Raw milk	242P ^a^	Raw milk	SA84 ^b^	Chicken
190P ^a^	Raw milk	243P ^a^	Raw milk	SA86 ^b^	Sausages
191P ^a^	Raw milk	244P ^a^	Raw milk	SA87 ^b^	Pastry
192P ^a^	Raw milk	367P ^a^	Raw milk	SA03 ^b^	Salad
193P ^a^	Raw milk	373P ^a^	Raw milk	SA04 ^b^	Meat
194P ^a^	Raw milk	374P ^a^	Raw milk	SA09 ^b^	Pastry
195P ^a^	Raw milk	200P ^a^	Raw milk	SA29 ^b^	Meat
196P ^a^	Raw milk	201P ^a^	Raw milk	SA31 ^b^	Salad
197P ^a^	Raw milk	211P ^a^	Raw milk	SA72 ^b^	Pastry
198P ^a^	Raw milk	212P ^a^	Raw milk	SA17 ^b^	Turkey pieces
199P ^a^	Raw milk	213P ^a^	Raw milk	SA46 ^b^	Braised beef
356P ^a^	Raw milk	214P ^a^	Raw milk	ATCC 27664 (FRI326) ^a^	Chicken tetrazzini
357P ^a^	Raw milk	234P ^a^	Raw milk	BS4 ^a^	“Napoli-type” salami
358P ^a^	Raw milk	SA01 ^b^	Potato in sauce	DS18g ^a^	“Napoli-type” salami
359P ^a^	Raw milk	SA06 ^b^	Chicken	AS14g ^a^	“Napoli-type” salami
360P ^a^	Raw milk	SA11 ^b^	Couscous with meat	382F ^a^	Unspecified food
361P ^a^	Raw milk	SA13 ^b^	Rice	ED-4 ^a^	Raw poultry meat
362P ^a^	Raw milk	SA41 ^b^	Salad	AB-8802 ^a^	Raw poultry meat

^a^ DNA extracted using the “classical method” (alcohol-based); isolates reported in Chieffi et al. [4], Fusco et al. [2], and Blaiotta et al. [29]. ^b^ DNA extracted using the “rapid method” (boiling in presence of a Chelex-based reagent); isolates reported in Mekhloufi et al. [30].

**Table 2:** Twenty-two clinical *S. aureus*, one *S. argenteus* isolate, and seven CoNS used in this study to verify the applicability of the developed rt-PCR-HRM assay at MMARLab (Catania, Italy).

Isolate	Species	MSSA/MRSA	ST
S1 2021	*S. argenteus*	-	-
ATCC 29213	*S. aureus*	MSSA	-
ATCC BAA-1556 (USA300)	*S. aureus*	MRSA	8
ATCC BAA-1707 (MW2/USA400)	*S. aureus*	MRSA	1
NRS385 (USA500)	*S. aureus*	MRSA	8
ATCC 700699 (MU50)	*S. aureus*	MRSA	5
NR-45906 (COL)	*S. aureus*	MRSA	250
ATCC BAA-44 (HPV107)	*S. aureus*	MRSA	247
CAUD	*S. aureus*	MRSA	88
ATCC BAA 2312-M10/0148	*S. aureus*	MRSA	130
HU25 (BEC)	*S. aureus*	MRSA	239
S2 2017	*S. aureus*	MRSA	111
S6 2017	*S. aureus*	MRSA	398
S1 2018	*S. aureus*	MRSA	30
S4 2018	*S. aureus*	MRSA	93
S5 2018	*S. aureus*	MRSA	2361
S1 2019	*S. aureus*	MRSA	8
S5 2019	*S. aureus*	MSSA	398
S2 2021	*S. aureus*	MRSA	22
S3 2021	*S. aureus*	MRSA	398
S1 2022	*S. aureus*	MRSA	121
S2 2022	*S. aureus*	MSSA	1
S3 2022	*S. aureus*	MSSA	152
ATCC 29971	*S. xylosus*	-	-
ATCC 27847	*S. hominis*	-	-
ATCC 14990	*S. epidermidis*	-	-
ATCC 27848	*S. simulans*	-	-
ATCC 49052	*S. intermedius*	-	-
ATCC 29970	*S. haemolyticus*	-	-
ATCC 27840	*S. capitis*	-	-

ST—sequence type.

**Table 3:** Mean melting temperatures (Tm) ± standard deviation (SD) and corresponding coefficients of variation (CV%) for 19 representative subset isolates tested in triplicate, in two independent runs performed on two different days, within the intra- and inter-assay reproducibility test.

Samples	Run 1		Run 2		Inter-Assay	
	Tm ± SD ^c^	CV%	Tm ± SD ^c^	CV%	Tm ± SD ^c^	CV%
**Reference Strains**						
*S. argenteus* DSM 28299^T^	75.94 ± 0.02	0.03	75.92 ± 0.04	0.05	75.93 ± 0.03	0.04
*S. aureus* DSM 20231^T^	77.24 ± 0.02	0.02	77.20 ± 0.04	0.05	77.22 ± 0.04	0.05
**Isolates**						
*S. aureus* (n = 13) ^a^	77.44 ± 0.05	0.06	77.25 ± 0.04	0.05	77.34 ± 0.11	0.14
*S. aureus* (n = 6) ^b^	77.12 ± 0.03	0.05	77.20 ± 0.03	0.04	77.16 ± 0.05	0.07
TOT *S. aureus* (n = 19) ^a,b^	77.34 ± 0.16	0.21	77.23 ± 0.04	0.05	77.28 ± 0.13	0.17

^a^ DNA extracted by “classical method” (alcohol-based DNA extraction). ^b^ DNA extracted by “rapid method” (DNA extraction by boiling in presence of a Chelex-based reagent). ^c^ Standard deviation.

**Table 4:** Limit of detection of the real-time PCR HRM tested during the assay development in the 7500 Fast Real-Time PCR System (Applied Biosystems, Waltham, MA, USA).

Reference Strains	Genome Equivalents	Ct ^a^	SD ^b^
*S. argenteus* DSM 28299^T^	10^7^	15.20	0.17
	10^6^	17.44	0.07
	10^5^	20.44	0.08
	10^4^	23.61	0.13
	10^3^	27.18	0.07
	10^2^	30.85	0.18
	10^1^	33.97	0.17
*S. aureus* DSM 20231^T^	10^7^	15.59	0.30
	10^6^	17.93	0.07
	10^5^	21.18	0.04
	10^4^	24.39	0.22
	10^3^	27.90	0.19
	10^2^	31.44	0.17
	10^1^	34.72	0.09

^a^ Threshold cycle. ^b^ Standard deviation.

## Data Availability

The original contributions presented in the study are included in the article/Supplementary Material, further inquiries can be directed to the corresponding author.

## Supplementary Materials

The following supporting information can be downloaded at https://www.mdpi.com/article/10.3390/foods13183004/s1, Figure S1: Derivative melt curves (a), normalized melt curves (b), and difference plot (c) generated by HRM analysis of the amplification products obtained for *S. aureus* DSM 20231^T^ (red), *S. argenteus* DSM 28299^T^ (green), and *S. schweitzeri* DSM 28300^T^ (blue) tested in triplicate within an intra-assay reproducibility test. Table S1: Mean melting temperatures (Tm) ± standard deviation (SD) and corresponding coefficients of variation (CV%) for *S. argenteus* DSM 28299^T^, *S. aureus* DSM 20231^T^, and *S. schweitzeri* DSM 28300^T^ tested in triplicate within an intra-assay reproducibility test. Figure S2: Amplification curves (a) and standard curve (b) of serial decimal dilutions (10^7^ to 10^1^ GE) of *S. aureus* DSM 20231^T^ DNA. Figure S3: Amplification curves (a) and standard curve (b) of serial decimal dilutions (10^7^ to 10^1^ GE) of *S. argenteus* DSM 28299^T^ DNA.
