# Supplementary material for: A Novel Approach Based on Real-Time PCR with High-Resolution Melting Analysis for the Simultaneous Identification of Staphylococcus aureus and Staphylococcus argenteus
Source: Foods. 2024 Sep 22;13(18):3004. doi: 10.3390/foods13183004 (PMC11431256; doi:10.3390/foods13183004)
Supplement: Supplementary file 1 [file foods-13-03004-s001.zip › foods-3200930-supplementary.pdf]

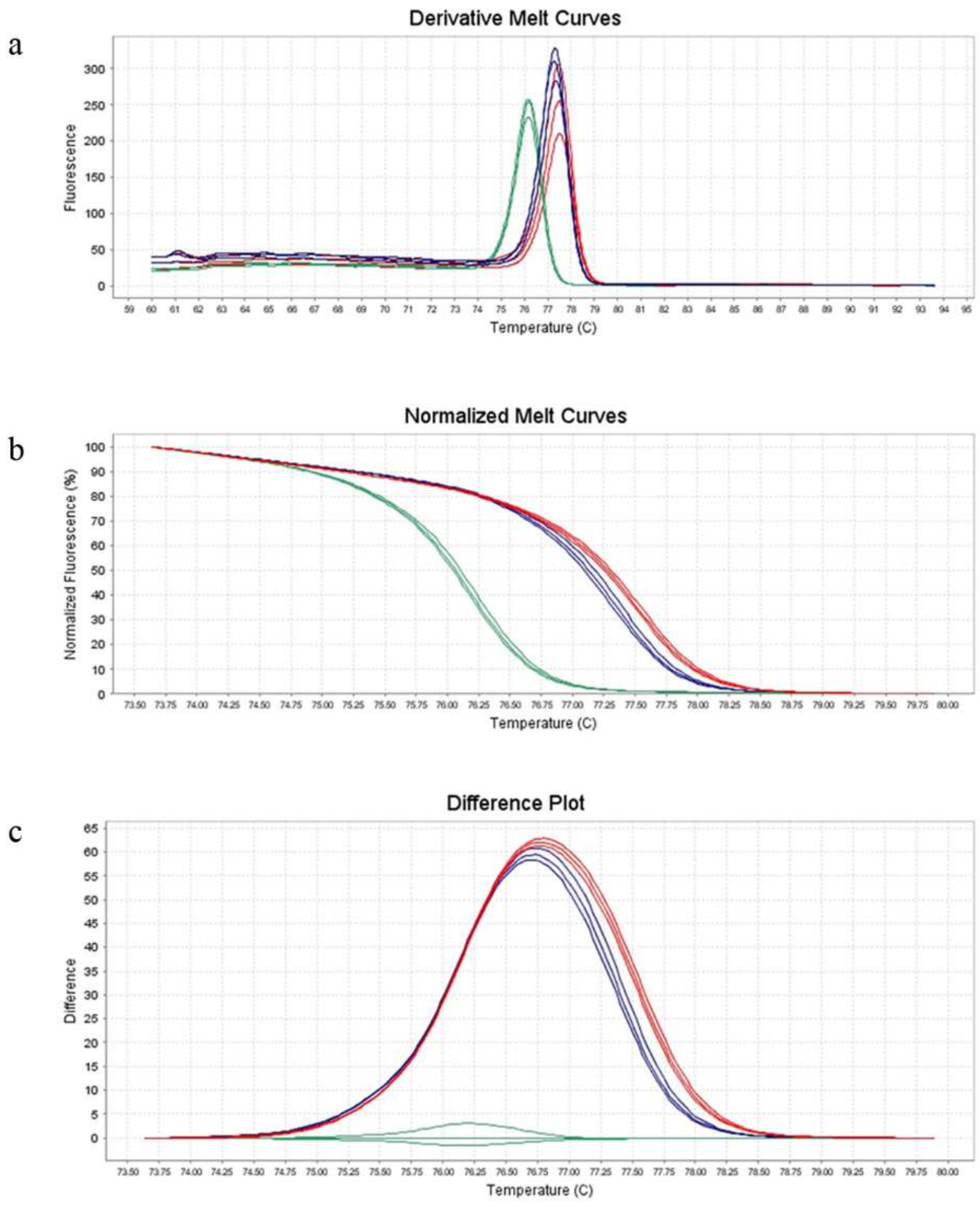

Figure S1. Derivative melt curves (a), normalized melt curves (b) and difference plot (c) generated by HRM analysis of the amplification products obtained for *S. aureus* DSM 20231^{T} (red), *S. argenteus* DSM 28299^{T} (green) and *S. schweitzeri* DSM 28300^{T} (blue) tested in triplicate within an intra-assay reproducibility test.

Table S1. Mean melting temperatures (Tm) ± standard deviation (SD), and corresponding coefficients of variation (CV%), for *S. argenteus* DSM 28299^{T}, *S. aureus* DSM 20231^{T} and *S. schweitzeri* DSM 28300^{T} tested in triplicate within an intra-assay reproducibility test.

Reference strains	Tm ± SD	CV%
S. argenteus DSM 28299^{T}	76.19 ± 0.02	0.03
S. aureus DSM 20231^{T}	77.48 ± 0.02	0.02
S. schweitzeri DSM 28300^{T}	77.31 ± 0.05	0.06

a

b

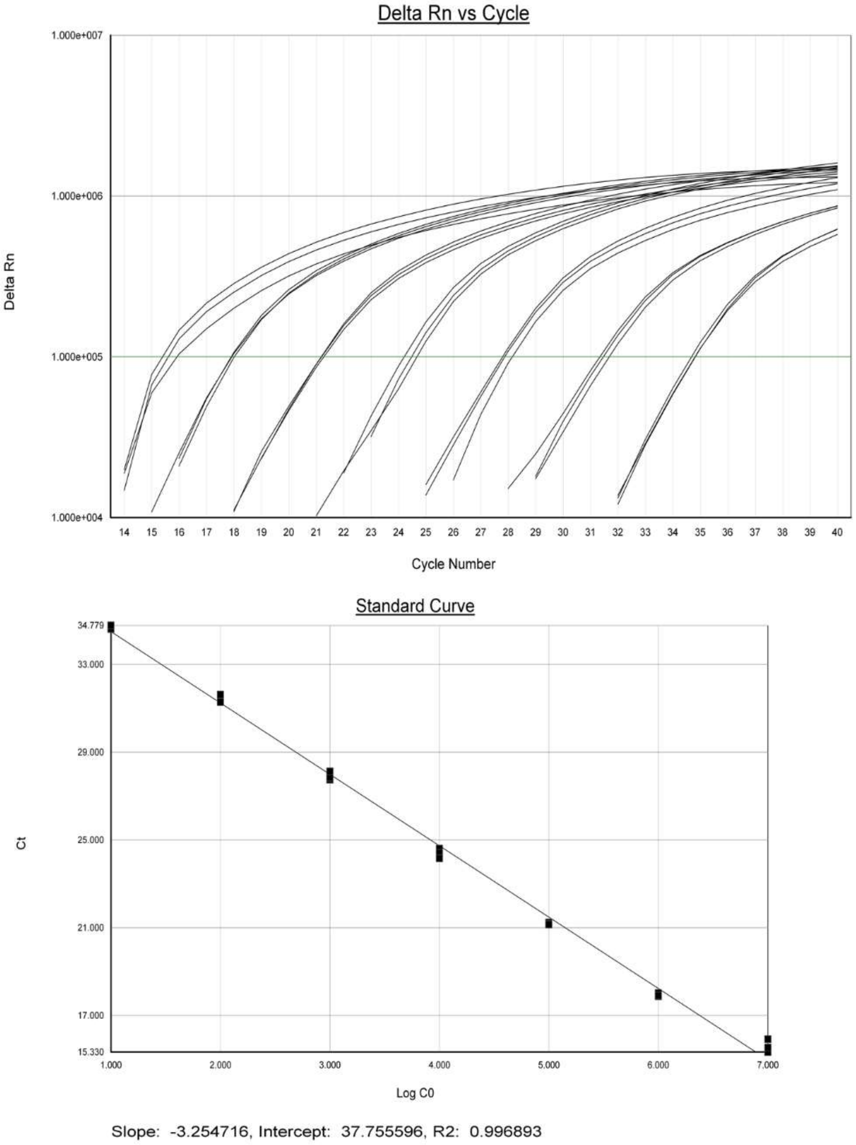

Figure S2. Amplification curves (a) and standard curve (b) of serial decimal dilutions (10^{7} to 10^{1} GE) of *S. aureus* DSM 20231^{T} DNA.

a

b

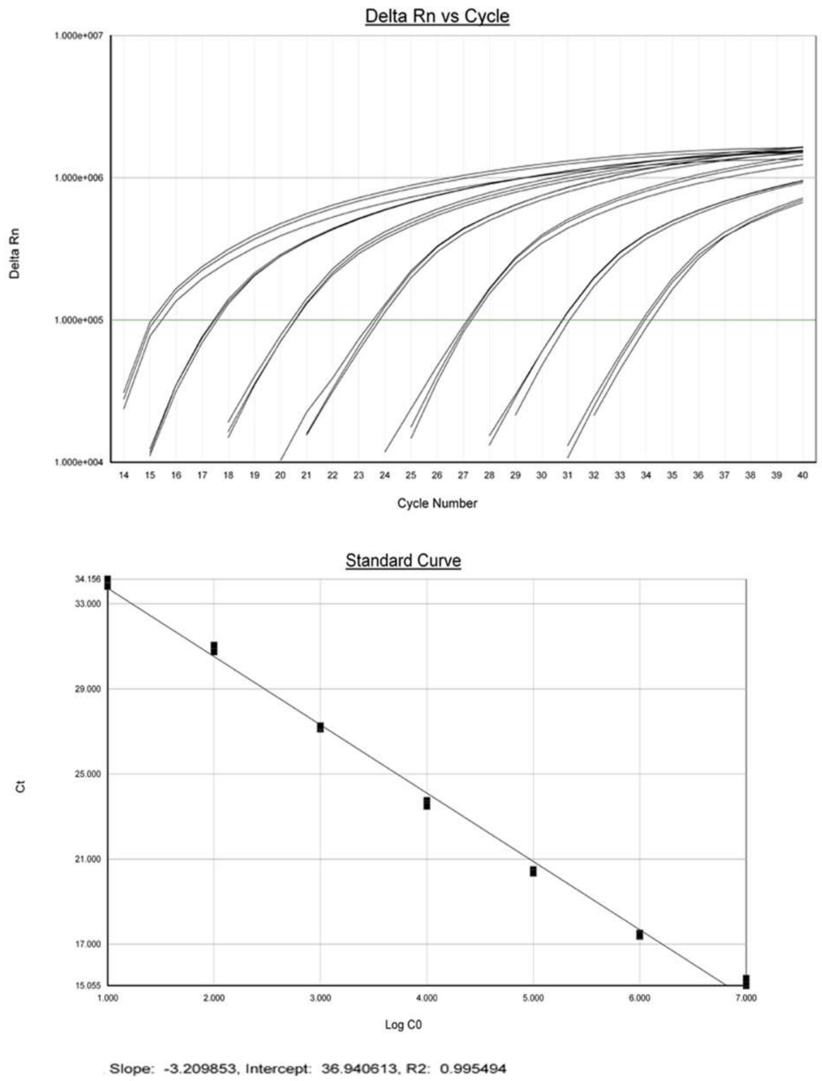

Figure S3. Amplification curves (a) and standard curve (b) of serial decimal dilutions (10^7 to 10^1 GE) of *S. argenteus* DSM 28299^T DNA.
